# Platelet-Rich Plasma Injections Do Not Improve the Recovery After Arthroscopic Partial Meniscectomy: A Double-Blind Randomized Controlled Trial

**DOI:** 10.1177/03635465241283052

**Published:** 2024-10-18

**Authors:** Mirco Lo Presti, Giuseppe Gianluca Costa, Giuseppe Agrò, Cosimo Vasco, Angelo Boffa, Alessandro Di Martino, Luca Andriolo, Annarita Cenacchi, Stefano Zaffagnini, Giuseppe Filardo

**Affiliations:** * Clinica Ortopedica e Traumatologica 2, IRCCS Istituto Ortopedico Rizzoli, Bologna, Italy; † U.O. Ortopedia e Traumatologia, Ospedale Umberto I, Enna, Italy; ‡ Istituto Clinico San Rocco, Ome, Brescia, Italy; § U.O.C. Ortopedia e Traumatologia, Ospedale S. Maria della Scaletta, Imola, Italy; ¶ Applied and Translational Research center (ATRc), IRCCS Istituto Ortopedico Rizzoli, Bologna, Italy; # Servizio Trasfusionale Unico Metropolitano, Bologna, Italy; ** Faculty of Biomedical Sciences, Università della Svizzera Italiana, Lugano, Switzerland; Investigation performed at IRCCS Istituto Ortopedico Rizzoli, Bologna, Italy

**Keywords:** knee, meniscus, meniscectomy, platelet-rich plasma, PRP

## Abstract

**Background:**

Arthroscopic meniscectomy is one of the most performed surgical procedures in orthopaedics. Different approaches have been proposed to improve patient recovery but with unsatisfactory results. Platelet-rich plasma (PRP) augmentation has been proposed as a strategy to improve the recovery after meniscectomy.

**Purpose:**

To investigate the clinical benefits of an intra-articular PRP injection after meniscectomy, in terms of faster and better patient recovery.

**Study Design:**

Randomized controlled trial; Level of evidence, 1.

**Methods:**

Ninety patients were randomized into a treatment group, with arthroscopic partial meniscectomy immediately followed by a 5-mL injection of autologous conditioned plasma, and a control group with partial meniscectomy alone. Patients were evaluated at baseline and at 15, 30, 60, and 180 days of follow-up with the visual analog scale (VAS) score for pain (primary outcome), as well as with International Knee Documentation Committee subjective score, Knee injury and Osteoarthritis Outcome Score subscales, Tegner score, and EuroQol-Visual Analog Scale score. Objective evaluation was performed analyzing knee range of motion and circumference and the International Knee Documentation Committee objective score. Complications, patient judgment, and satisfaction were documented as well.

**Results:**

No major complications and an overall significant improvement in the clinical scores were observed in both groups. Overall, the comparative analysis did not demonstrate significant between-group differences in absolute values or improvements of both subjective and objective scores, as well as activity level. The improvement in terms of VAS pain score for the treatment group was significant already at 15 days (from 4.3 ± 2.5 to 2.5 ± 2.5; *P* = .014), while in the control group it became significant at 30 days (from 3.7 ± 2.3 to 2.0 ± 2.4; *P* = .004). No significant differences were observed between the 2 groups in terms of judgment of treatment results and satisfaction.

**Conclusion:**

A single postoperative injection of PRP was not able to significantly improve patient recovery after arthroscopic partial meniscectomy. PRP augmentation did not provide overall benefits at a short-term follow-up (6 months) in terms of pain relief, function, objective parameters, and return-to-sport activities.

**Registration:**

NCT02872753 (ClinicalTrials.gov identifier).

Meniscal tears represent common knee injuries addressed by nonsurgical and surgical approaches, such as arthroscopic meniscectomy and meniscal repair.^1,3,11,13,15,25,26^ Despite the increasing interest in the “save the meniscus” concept through meniscal repair, arthroscopic meniscectomy continues to be necessary for various types of nonrepairable injuries.^4,29,31^ Therefore, this treatment still represents one of the most performed surgical procedures in orthopaedic clinical practice, offering low postoperative morbidity and a relatively rapid recovery. However, recovery after meniscectomy can be affected by alterations resulting from both knee trauma and surgical insult, which can alter the joint homeostasis via the release of catabolic molecules and proinflammatory factors.^21^ To minimize the postoperative discomfort and improve patient recovery after meniscectomy, different approaches have been investigated, from oral medications to intra-articular injections of steroids or hyaluronic acid, but with unsatisfactory results.^17,24^

The use of orthobiologics like platelet-rich plasma (PRP) has been recently proposed as a simple and minimally invasive treatment strategy to improve the recovery and clinical outcomes after meniscectomy, acting directly on the meniscal tissue and indirectly by restoring or improving joint homeostasis.^8,18^ In particular, PRP exploits the high concentrations of cytokines and growth factors stored in platelet α-granules, which have been shown to take part in the homeostasis of articular cartilage involved in both the healing process and immunoregulation.^18^ These biologically active proteins can influence and promote a more physiological joint environment, favoring the restoration of a homeostatic balance in the joint.^8^ In this light, preclinical studies on osteoarthritis models induced via meniscectomy have demonstrated the ability of PRP to slow down the progression of cartilage damage and articular inflammatory processes.^8^ Moreover, in vitro studies have documented positive effects of PRP on meniscal cells, upregulating their viability in a dose-dependent manner, as well as favoring the mRNA expression of biglycan and decorin.^23^ Therefore, the use of PRP after meniscectomy could potentially promote a faster recovery, although clinical evidence on this orthobiologic approach is still limited.

The aim of this double-blind randomized controlled trial (RCT) was to investigate the clinical benefits in terms of an earlier patient recovery provided by an intra-articular PRP injection after arthroscopic partial meniscectomy.

## Methods

### Patient Selection and Study Design

This prospective double-blind RCT was approved by the hospital ethics committee of the IRCCS Istituto Ortopedico Rizzoli, Bologna, Italy (protocol No. 0016969). The trial was registered at ClinicalTrials.gov (registration No. NCT02872753) and was entirely conducted in a highly specialized referral center for orthopaedic pathologies. Patients were enrolled and treated between July 2017 and June 2022 and were considered eligible for study inclusion if they met the following criteria: aged between 18 and 55 years, had symptomatic (>1 month) isolated meniscal tears (pain and/or mechanical symptoms and positive meniscal tests), evaluated using magnetic resonance imaging, required partial resection, and had a healthy contralateral knee (ie, no pain or functional limitation in the contralateral joint). The following exclusion criteria were used: meniscal lesions requiring suture; previous surgery on the index knee; other concurrent articular lesions (eg, cartilage or ligament injuries) requiring surgical treatment (eg, chondroplasty or other cartilage procedures, ligament reconstructions); history of knee septic arthritis; concurrent rheumatic, metabolic, hematological, cardiovascular, or severe systemic diseases; body mass index >30; anticoagulant or antiaggregant therapy; use of nonsteroidal anti-inflammatory drugs in the 3 days before blood harvest; hemoglobin level of <11 g/dL or platelet count of <150,000/mm^3^ at blood harvest; alcohol or other substance abuse; and patient incapacitated or otherwise unable to provide consent. Screening, enrollment, and informed consent for study participation were obtained from each patient at the time of admission in the institute.

**Figure 1.**
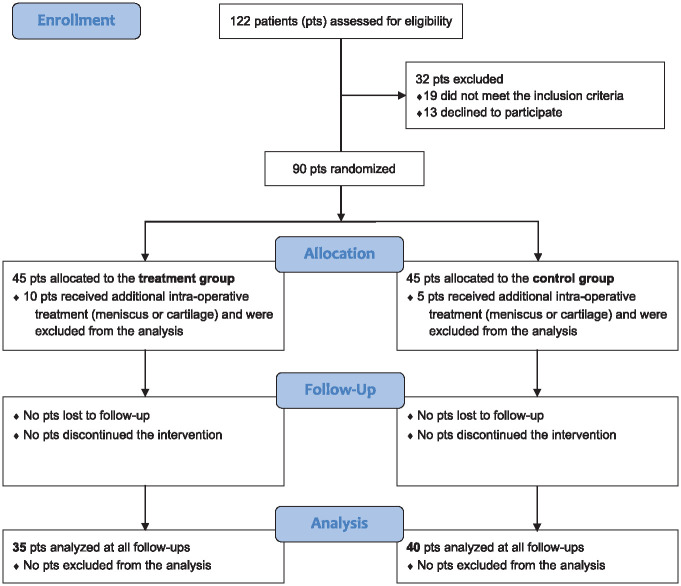
CONSORT (Consolidated Standards of Reporting Trials) flow diagram used in the design of the trial. pts, patients.

A total of 90 consecutive patients affected by isolated meniscal tears requiring partial resection met the inclusion criteria and were included in this study. The CONSORT (Consolidated Standards of Reporting Trials) flow diagram is shown in Figure 1. Patients were randomly assigned to 2 groups at a 1:1 ratio using a computer-generated randomization system: the treatment group underwent arthroscopic partial meniscectomy followed by an injection of autologous conditioned plasma (ACP; Arthrex), and the control group underwent arthroscopic partial meniscectomy alone. The randomization list (block randomization with block sizes of 10 patients) was provided by an independent statistician, generated using a random number generator, and then kept in a dedicated office. Allocation to the treatment or control group was kept concealed from the patient until the end of the study. Moreover, the clinicians who assessed the patients at follow-up (G.G.C., G.A, and C.V.) were unaware of the randomization and treatment allocation.

### Treatment Procedures

Treatment was performed at a specialized orthopaedic institute by orthopaedic surgeons (S.Z., M.L.P., and A.D.M.) with vast experience in sports medicine and orthobiologics. Before the arthroscopic surgery, all patients underwent venous blood collection at the transfusion unit of the same institute to maintain the blinding to group allocation. In detail, 15 mL of peripheral venous blood was drawn using a double-syringe system and subsequently processed via centrifugation at 1500 rpm for 5 minutes to obtain 5 mL of ACP, a single-spin leukocyte-poor PRP. The arthroscopic partial meniscectomy procedure was performed in a single step in the operating room with patients in the supine position and according to the hospital protocol: the procedure involved, after transient limb ischemia, creating standard anteromedial and anterolateral arthroscopic access to remove the damaged part of the meniscus, preserving its integrity as much as possible. At the end of the surgical procedure, based on the randomization list, half of the patients received an injection of the obtained ACP, and the other half did not. ACP was not associated with the use of anesthetics or corticosteroids, and it was administered through the arthroscopic portal after the portal was sutured, with the patient still covered by the surgical drape, to maintain patient blinding.

The postoperative protocol was the same for all included patients, including the application of local ice and elevation of the operated limb in the hours after surgery. Then, patients were discharged on the same day of surgery with specific indications for rehabilitation consisting of progressive weightbearing using 2 crutches for 2 weeks, gradual recovery of passive and active range of motion (ROM), and muscle strengthening with isometric exercises. Subsequently, patients were encouraged to gradually return to daily and sport activities as tolerated.

### Clinical Outcomes

All follow-up visits were performed by physicians not involved in the surgical procedure to ensure double blinding. Patients were evaluated at baseline and at 15, 30, 60, and 180 days postoperatively. The primary outcome was defined as the change in the visual analog scale (VAS) score for pain from baseline to 30 days after the procedure. The VAS score was also evaluated at 15, 60, and 180 days of follow-up. Other subjective scores were used to complete patient evaluation, including the International Knee Documentation Committee (IKDC) subjective score, the Knee injury and Osteoarthritis Outcome Score (KOOS) subscales, the Tegner score for the sport/activity level, and the EuroQol-Visual Analog Scale score at 30, 60, and 180 days of follow-up. Moreover, an objective evaluation was performed at 15, 30, and 60 days after treatment through measurement of knee ROM using a goniometer, knee circumference at the patellar center level using a ruler, and the IKDC objective score. Finally, patient judgment of the treatment and satisfaction were collected at the last follow-up. Patient judgment of the treatment was evaluated using a specific question: “Compared to the baseline status, how would you rate the treated knee now?” The response was recorded using a 6-point scale: “full recovery,”“much better,”“somewhat better,”“about the same,”“somewhat worse,” and “much worse.” Patient satisfaction was evaluated using a specific question: “Are you satisfied with your knee health status?” The response was recorded using a 2-point scale: “satisfied” or “not satisfied.”

All complications and adverse events were assessed and reported during the follow-up visits. The treatment was considered to have failed if the patient needed another injection or surgical treatment procedure because of symptom persistence or worsening. For treatment failure, the patient's worst clinical evaluation between baseline and the last available follow-up was considered for the following assessments.

### Statistical Analysis

For the sample size calculation, a power analysis was performed for improvement in the primary outcome (VAS pain score) at 30 days. A previous study revealed a standard deviation of 1.3 points. With an alpha error of .05 and assuming a minimal clinically important difference of 1 point, the minimum sample size was 74 patients. To account for a possible dropout rate of 20%, 45 patients per group, for a total of 90 patients, were required for the study purpose. All continuous data are expressed as mean and standard deviation, and categorical data are expressed as frequency and percentage. The Shapiro-Wilk test was performed to test the normality of continuous variables. The Levene test was used to assess the homoscedasticity of the data. The repeated-measures general linear model with Sidak test for multiple comparisons was performed to assess the differences at different follow-up times of the quantitative scores. The Friedman nonparametric test followed by Wilcoxon signed rank pairwise comparisons with Bonferroni correction was performed to assess the differences at different follow-up times of the ordinal scores. One-way analysis of variance with Scheffé post hoc pairwise analysis was performed to assess differences among groups when the Levene test for homogeneity of variances was not significant (*P* < .05); otherwise, the Mann-Whitney U test (2 groups) or the Kruskal-Wallis test with the nonparametric post hoc pairwise Dunnett test was used. The Spearman rank correlation was used to assess correlations between quantitative scores and continuous data. The Kendall tau correlation was used to assess correlations between quantitative or ordinal scores and ordinal data. The Fisher chi-square exact test was performed to assess the relationship between dichotomous variables. The Pearson chi-square test evaluated using the Fisher exact test was performed to investigate the relationship between categorical variables. For all tests, *P* < .05 was considered significant. All statistical analysis was performed using SPSS Version 19.0 (IBM Corp).

## Results

The study population included 75 patients. In detail, 90 consecutive patients were randomly assigned to the treatment group (45 patients) or control group (45 patients). Among these, 15 patients were excluded from the analysis because they underwent a different treatment than partial meniscectomy (eg, meniscal repair) or an unplanned associated procedure (ie, microfracture or chondroplasty), confirmed after performing the arthroscopy. Thus, 35 patients in the treatment group and 40 patients in the control group were included in the study. As reported in Table 1, the 2 groups were homogeneous for all baseline characteristics, except for preoperative ROM.

**Table 1:** Baseline Characteristics of the Included Patients^*a*^

	Treatment Group (ACP Injection After Meniscectomy)	Control Group (Meniscectomy Alone)
Sex, male/female	28/7	31/9
Age, y	38.6 ± 7.8	39.4 ± 8.2
BMI	24.8 ± 2.6	24.3 ± 2.9
Side, left/right	22/13	20/20
Meniscus involved, med/lat/both	29/5/1	30/9/1
Symptom duration, mo, mean (range)	13.5 (2-60)	16.3 (3-100)
VAS pain score	4.3 ± 2.5	3.7 ± 2.3
IKDC subjective score	47.4 ± 14.0	49.7 ± 17.9
KOOS Pain	62.5 ± 18.3	65.5 ± 20.3
KOOS Symptoms	66.2 ± 17.1	69.0 ± 20.2
KOOS ADL	75.7 ± 16.9	75.2 ± 22.6
KOOS Sport/Rec	49.0 ± 23.0	53.7 ± 24.1
KOOS QoL	39.7 ± 21.2	39.8 ± 20.1
EQ-VAS score	78.3 ± 11.9	80.0 ± 12.7
Tegner preinjury score	4.7 ± 1.4	4.6 ± 1.6
Tegner pretreatment score	2.7 ± 1.3	2.9 ± 1.7
IKDC objective score	1.4 ± 0.9	1.9 ± 1.1
ROM, deg	135.1 ± 14.2	127.4 ± 17.2
Knee circumference, mm	376.6 ± 32.5	368.0 ± 27.0

a Data are presented as number or mean ± SD unless otherwise indicated. ACP, autologous conditioned plasma; ADL, Activities of Daily Living; BMI, body mass index; both, medial and lateral menisci; EQ-VAS, EuroQol–Visual Analog Scale; IKDC, International Knee Documentation Committee; KOOS, Knee injury and Osteoarthritis Outcome Score; lat, lateral meniscus; med, medial meniscus; QoL, Quality of Life; ROM, range of motion; Sport/Rec, Sport/Recreation; VAS, visual analog scale.

No major complications and adverse events were reported in either group during the follow-up evaluation. One patient (treatment group) experienced treatment failure at 30 days of follow-up due to knee swelling requiring joint aspiration and subsequent intra-articular corticosteroid injection.

**Figure 2.**
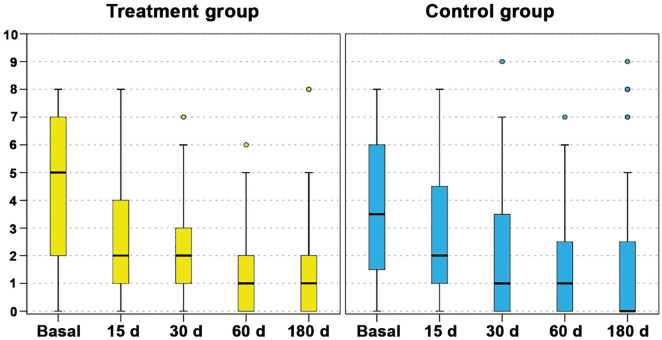
The visual analog scale for pain trend in the treatment group (left) and control group (right) at baseline and at 15, 30, 60, and 180 days of follow-up. The box-and-whisker plots show the median (whiskers), interquartile range (boxes), and 95% confidence interval (lines). No differences were found between treatment and control groups in terms of absolute values and improvement from baseline.

An overall significant improvement in the clinical scores was observed in both groups. The comparative analysis of the primary outcome did not demonstrate any significant differences between the 2 groups in terms of absolute values and improvement from baseline (Figure 2). The only statistically significant differences between the treatment group and the control group were observed in terms of absolute score values documented for KOOS Sport/Recreation at 60 days (69.3 ± 22.2 and 79.3 ± 21.7, respectively; *P* = .039) and KOOS Quality of Life at 60 days (64.7 ± 25.7 and 76.6 ± 22.1, respectively; *P* = .033), although the comparison of the improvements from baseline to the same follow-up in the 2 groups did not confirm a significant difference. In the treatment group, the VAS pain score significantly improved from the baseline value of 4.3 ± 2.5 to 2.5 ± 2.5 at 15 days (*P* = .014), 2.0 ± 1.7 at 30 days (*P* < .0005), 1.5 ± 1.8 at 60 days (*P* < .0005), and 1.6 ± 2.2 at 180 days (*P* < .0005) of follow-up. In the control group, the VAS pain score significantly improved, although the improvement was not statistically significant from the baseline value of 3.7 ± 2.3 to 2.7 ± 2.3 at 15 days, whereas it was statistically significant from baseline to 30 days (2.0 ± 2.4; *P* = .004), 60 days (1.4 ± 1.9; *P* < .0005), and 180 days (1.8 ± 2.9; *P* = .012). The other subjective scores demonstrated similar trends with statistically significant improvements from baseline to all follow-ups in both groups, as reported in Appendix Table A1 (available in the online version of this article).

Regarding the sport activity, the Tegner score significantly improved in the treatment group from the preoperative mean value of 2.7 ± 1.3 to 3.6 ± 1.2 at 60 days (*P* = .033) and 4.3 ± 1.6 at 180 days (*P* < .0005), reaching the preinjury value (4.7 ± 1.4) at the last follow-up. In the control group, the Tegner score improved significantly from the preoperative mean value of 2.9 ± 1.7 to 3.9 ± 1.5 only at 180 days (*P* = .040), without reaching the preinjury value (4.6 ± 1.6; *P* = .044) at the last follow-up. At the last follow-up, 51.4% of patients in the treatment group reached the same sport activity level practiced before the injury, compared with 45.0% in the control group, without statistically significant differences between the 2 groups.

Regarding the objective evaluations, ROM significantly worsened from baseline to 15 days of follow-up in the treatment group (from 135.1°± 14.2° to 112.1°± 16.8°; *P* < .0005) and in the control group (from 127.4°± 17.2° to 113.5°± 19.4°; *P* = .006), and it returned to values comparable to baseline in both the treatment and control groups at 30 (128.1°± 15.7° and 128.0°± 15.7°, respectively) and 60 days (136.1°± 17.0° and 134.1°± 5.0°, respectively) of follow-up. The analysis of the IKDC objective score showed a nonsignificant improvement from baseline to all follow-up evaluations in both groups. At the baseline evaluation, 31 knees (88.6%) in the treatment group and 38 knees (95.0%) in the control group were considered normal or nearly normal, compared with 33 knees (94.3%) in the treatment group and 39 knees (97.5%) in the control group at 60 days. Finally, no statistically significant changes were observed in knee circumference from baseline to all follow-ups in both groups.

In the treatment group, at the final follow-up, 32 patients (94.1%) rated their knee as improved (12 as full recovery, 17 as much better, 3 as somewhat better), and 2 patients reported no change or slight worsening after treatment. Moreover, 30 patients (88.2%) were satisfied with the treatment. In the control group, at the final follow-up, all patients rated their knee as improved (12 as full recovery, 21 as much better, 7 as somewhat better) and 39 patients (97.5%) were satisfied with the treatment. No statistically significant differences were observed between the 2 groups in terms of judgment of treatment results and satisfaction.

Age, sex, body mass index, knee side, involved meniscus, and symptom duration did not influence the clinical outcomes in either the treatment or the control group.

## Discussion

The main finding of this double-blind RCT is that a single postoperative injection of PRP was not able to significantly improve patient recovery after arthroscopic partial meniscectomy. PRP surgery augmentation did not provide overall benefits in terms of pain relief, function, objective parameters, and return-to-sport activities.

Research efforts aim to improve recovery after arthroscopic meniscectomy by targeting the joint inflammatory status postinjury and postsurgery. In fact, meniscal tears are detrimental not only to knee biomechanics but also to joint homeostasis. The surgical insult caused by meniscectomy can further promote an inflammatory joint environment, affecting the postoperative recovery. Ogura et al^27^ reported increased levels of proinflammatory cytokines in patients with injured meniscal tissue, and Scanzello et al^30^ observed synovial biopsy specimens with increased inflammation in almost 1 of 2 patients treated for meniscal injury, associated with worse preoperative pain and functional score. Thus, treating homeostasis alterations by reducing joint inflammation could promote a faster recovery and improve clinical outcomes after arthroscopic partial meniscectomy.

Corticosteroid injections have been proposed to improve the postmeniscectomy outcome through their well-known anti-inflammatory potential.^12^ The current literature suggests that intra-articular corticosteroid injections at the time of diagnostic arthroscopy or meniscectomy decrease postoperative pain scores, analgesic use, and recovery time.^7,24^ Nevertheless, more recent evidence has emphasized that corticosteroid administration before or after knee arthroscopy can increase the risk of reoperation and infection.^5,9,10,19,20^ Hyaluronic acid has been suggested as an alternative to improve the altered joint environment after arthroscopic meniscectomy, promoting postoperative pain relief and expediting functional improvement.^2,32^ However, the administration of a single hyaluronic acid injection at the end of the surgical procedure does not provide either benefit, as demonstrated in a double-blind RCT on 90 patients affected by symptomatic meniscal tears requiring partial resection.^17^ Thus, research attention has been directed toward the use of orthobiologics such as PRP to exploit their therapeutic potential for improving recovery after arthroscopic meniscectomy.

PRP is a fashionable option for patients undergoing arthroscopic meniscectomy because of its ability to deliver a high concentration of growth factors and cytokines involved in both the meniscal tissue healing process and joint environment.^8,18,23^ PRP has shown a significant inflammation modulatory activity, decreasing inflammatory molecule expression, preventing chemotaxis of monocyte-like cells, and overall promoting a better joint homeostatic balance.^8,18,22^ However, despite the increasing interest in the application of this orthobiologic in the orthopaedic clinical practice, evidence on its use after meniscectomy is still limited. A recent retrospective clinical study conducted by Duru et al^16^ analyzed 40 patients receiving a single intra-articular leukocyte-poor PRP injection after arthroscopic meniscectomy, comparing their results at 3, 6, and 12 months of follow-up with those obtained in 49 patients not receiving biological augmentation after surgery. The authors reported better clinical outcomes at 3 months and a lower reoperation rate at the final follow-up in the PRP group compared with the control group. Nevertheless, PRP augmentation did not provide overall benefits in terms of pain relief compared with baseline, and no advantages have been recorded at longer follow-up. Therefore, solid conclusions on the indication for using PRP after arthroscopic meniscectomy have not been reached.

The current study was designed as a high-level double-blind RCT to clarify the controversies remaining on this topic, analyzing the clinical advantages of a PRP with a low concentration of platelets and leukocytes injected after arthroscopic partial meniscectomy compared with surgery alone. The intra-articular injection at the end of the arthroscopic meniscectomy provided some benefits to the surgical procedure. Patients in the treatment group showed a significant improvement in terms of pain relief (VAS pain score) starting from the earliest 15-day follow-up, while the improvement became significant in the control group only after 30 days. Similarly, the PRP group showed a significant improvement in terms of sport activity level (Tegner score) at 60 days of follow-up, while the improvement became significant in the control group only at 180 days of follow-up. Moreover, the sport activity level at 180 days reached the same preinjury level in the PRP group, while it remained lower compared with the preinjury level in the control group. However, despite these advantages provided by the orthobiologic augmentation, direct comparison between the 2 groups did not show overall significant differences in the scores at the different follow-up evaluations. Instead, the only statistically significant differences between the 2 groups were observed in favor of the control group in terms of absolute score values documented for KOOS Sport/Recreation and KOOS Quality of Life at 60 days of follow-up, although these differences were not confirmed by comparison of the improvement obtained in the 2 groups from baseline to the same follow-up. Accordingly, the suggested benefits of intra-articular PRP injection to arthroscopic partial meniscectomy should be considered not significant and not clinically relevant compared with the improvement provided by the arthroscopic procedure alone. Based on the results of this RCT, a single PRP injection as augmentation after partial meniscectomy is not supported for clinical practice.

The findings of this RCT are strengthened by the high methodological quality of the study design. The double-blind investigation minimizes the risk of various types of biases, such as observer bias or confirmation bias, and also protects from the high-placebo effect characterizing orthobiologic and injectable treatments.^28^ The homogeneity of the 2 study groups at baseline represents another strength of this trial. This homogeneity was maintained during the entire follow-up period evaluation by excluding from the analysis all patients evaluated with other concurrent articular lesions or surgical procedures different from arthroscopic partial meniscectomy, to avoid any confounding factor. Nevertheless, there are some limitations that should be considered. This study analyzed the effects of a specific PRP with low concentrations of platelet and leukocytes, not providing information on the potential of different PRP formulations. Although the current literature seems to exclude an influencing role of leukocytes in the safety and efficacy of PRP injections,^14^ recent evidence has suggested that platelet concentration may lead to different outcomes in PRP treatment.^6^ Moreover, this RCT investigated a single administration of PRP, without exploring different injection protocols that could lead to a different result. Therefore, future studies should investigate whether augmentation with different PRP formulations and different injection protocols could have a different effect on patient recovery after arthroscopic partial meniscectomy.

## Conclusion

This double-blind RCT demonstrated that a single postoperative injection of PRP was not able to significantly improve patient recovery after arthroscopic partial meniscectomy. PRP augmentation did not provide overall benefits in terms of pain relief, function, objective parameters, and return-to-sport activities.

## Supplemental Material

sj-pdf-1-ajs-10.1177_03635465241283052 – Supplemental material for Platelet-Rich Plasma Injections Do Not Improve the Recovery After Arthroscopic Partial Meniscectomy: A Double-Blind Randomized Controlled Trial Supplemental material, sj-pdf-1-ajs-10.1177_03635465241283052 for Platelet-Rich Plasma Injections Do Not Improve the Recovery After Arthroscopic Partial Meniscectomy: A Double-Blind Randomized Controlled Trial by Mirco Lo Presti, Giuseppe Gianluca Costa, Giuseppe Agrò, Cosimo Vasco, Angelo Boffa, Alessandro Di Martino, Luca Andriolo, Annarita Cenacchi, Stefano Zaffagnini and Giuseppe Filardo in The American Journal of Sports Medicine

## References

[1] Adams BG Houston MN Cameron KL. The epidemiology of meniscus injury. Sports Med Arthrosc Rev. 2021;29(3):e24-e33. 10.1097/JSA.0000000000000329 34398119

[2] Altman RD Manjoo A Fierlinger A Niazi F Nicholls M. The mechanism of action for hyaluronic acid treatment in the osteoarthritic knee: a systematic review. BMC Musculoskelet Disord. 2015;16:321. 26503103 10.1186/s12891-015-0775-z PMC4621876

[3] Bahns C Bolm-Audorff U Seidler A Romero Starke K Ochsmann E. Occupational risk factors for meniscal lesions: a systematic review and meta-analysis. BMC Musculoskelet Disord. 2021;22(1):1042. 34911509 10.1186/s12891-021-04900-7 PMC8672613

[4] Beaufils P Pujol N. Management of traumatic meniscal tear and degenerative meniscal lesions. Save the meniscus. Orthop Traumatol Surg Res. 2017;103(8S):S237-S244. 10.1016/j.otsr.2017.08.003 28873348

[5] Belk JW Keeling LE Kraeutler MJ , et al. Risk of infection in knee arthroscopy patients undergoing corticosteroid injections in the perioperative period. Orthop J Sports Med. 2021;9(8):23259671211032941. 10.1177/23259671211032941 PMC8375342 34423063

[6] Bensa A Previtali D Sangiorgio A Boffa A Salerno M Filardo G. PRP injections for the treatment of knee osteoarthritis: the improvement is clinically significant and influenced by platelet concentration. A meta-analysis of randomized controlled trials. Am J Sports Med. 2024. doi:10.1177/03635465241246524. 10.1177/03635465241246524 PMC11874499 39751394

[7] Bhattacharjee DP Biswas C Haldar P Ghosh S Piplai G Rudra JS. Efficacy of intraarticular dexamethasone for postoperative analgesia after arthroscopic knee surgery. J Anaesthesiol Clin Pharmacol. 2014;30(3):387-390. 25190949 10.4103/0970-9185.137273 PMC4152681

[8] Boffa A Salerno M Merli G , et al. Platelet-rich plasma injections induce disease-modifying effects in the treatment of osteoarthritis in animal models. Knee Surg Sports Traumatol Arthrosc. 2021;29(12):4100-4121. 34341845 10.1007/s00167-021-06659-9

[9] Cancienne JM Gwathmey FW Werner BC. Intraoperative corticosteroid injection at the time of knee arthroscopy is associated with increased postoperative infection rates in a large Medicare population. Arthroscopy. 2016;32(1):90-95. 26553960 10.1016/j.arthro.2015.09.003

[10] Cancienne JM Werner BC Luetkemeyer LM Browne JA. Does timing of previous intra-articular steroid injection affect the post-operative rate of infection in total knee arthroplasty? J Arthroplasty. 2015;30(11):1879-1882. 26071248 10.1016/j.arth.2015.05.027

[11] Chirichella PS Jow S Iacono S Wey HE Malanga GA. Treatment of knee meniscus pathology: rehabilitation, surgery, and orthobiologics. PM R. 2019;11(3):292-308. 30195704 10.1016/j.pmrj.2018.08.384

[12] Coutinho AE Chapman KE. The anti-inflammatory and immunosuppressive effects of glucocorticoids, recent developments and mechanistic insights. Mol Cell Endocrinol. 2011;335(1):2-13. 20398732 10.1016/j.mce.2010.04.005 PMC3047790

[13] Deviandri R Daulay MC Iskandar D Kautsar AP Lubis AMT Postma MJ. Health-economic evaluation of meniscus tear treatments: a systematic review. Knee Surg Sports Traumatol Arthrosc. 2023;31(9):3582-3593. 36637478 10.1007/s00167-022-07278-8 PMC10435400

[14] Di Martino A Boffa A Andriolo L , et al. Leukocyte-rich versus leukocyte-poor platelet-rich plasma for the treatment of knee osteoarthritis: a double-blind randomized trial. Am J Sports Med. 2022;50(3):609-617. 35103547 10.1177/03635465211064303

[15] Di Paola J . Disability, impairment, and physical therapy utilization after arthroscopic partial meniscectomy in patients receiving workers’ compensation. J Bone Joint Surg Am. 2012;94(6):523-530. 22438001 10.2106/JBJS.K.00076

[16] Duru N Williams G Jr Assid E Renshaw A Jones D. Comparative, controlled, retrospective study of patient-reported outcomes after meniscectomy with adjunctive use of platelet-rich plasma or amniotic umbilical cord tissue. Ochsner J. 2024;24(1):6-13. 38510228 10.31486/toj.23.0073 PMC10949044

[17] Filardo G Di Matteo B Tentoni F , et al. No effects of early viscosupplementation after arthroscopic partial meniscectomy: a randomized controlled trial. Am J Sports Med. 2016;44(12):3119-3125. 27528611 10.1177/0363546516660070

[18] Filardo G Kon E Roffi A Di Matteo B Merli ML Marcacci M. Platelet-rich plasma: why intra-articular? A systematic review of preclinical studies and clinical evidence on PRP for joint degeneration. Knee Surg Sports Traumatol Arthrosc. 2015;23(9):2459-2474. 24275957 10.1007/s00167-013-2743-1 PMC4541701

[19] Forsythe B Berlinberg EJ Forlenza EM Oeding JF Patel HH Mascarenhas R. Corticosteroid injections 2 months before arthroscopic meniscectomy increases the rate of postoperative infections requiring surgical irrigation and debridement. Knee Surg Sports Traumatol Arthrosc. 2022;30(11):3796-3804. 35622120 10.1007/s00167-022-06981-w

[20] Forsythe B Forlenza EM Agarwalla A , et al. Corticosteroid injections 1 month before arthroscopic meniscectomy increase the risk of surgical-site infection. Arthroscopy. 2021;37(9):2885-2890 e2882. 10.1016/j.arthro.2021.02.047 33812029

[21] Heard BJ Barton KI Chung M , et al. Single intra-articular dexamethasone injection immediately post-surgery in a rabbit model mitigates early inflammatory responses and post-traumatic osteoarthritis-like alterations. J Orthop Res. 2015;33(12):1826-1834. 26135713 10.1002/jor.22972

[22] Huang G Hua S Yang T Ma J Yu W Chen X. Platelet-rich plasma shows beneficial effects for patients with knee osteoarthritis by suppressing inflammatory factors. Exp Ther Med. 2018;15(3):3096-3102. 29599843 10.3892/etm.2018.5794 PMC5867452

[23] Ishida K Kuroda R Miwa M , et al. The regenerative effects of platelet-rich plasma on meniscal cells in vitro and its in vivo application with biodegradable gelatin hydrogel. Tissue Eng. 2007;13(5):1103-1112. 17348798 10.1089/ten.2006.0193

[24] Koyonos L Yanke AB McNickle AG , et al. A randomized, prospective, double-blind study to investigate the effectiveness of adding depomedrol to a local anesthetic injection in postmeniscectomy patients with osteoarthritis of the knee. Am J Sports Med. 2009;37(6):1077-1082. 19279226 10.1177/0363546508331204

[25] Luvsannyam E Jain MS Leitao AR Maikawa N Leitao AE. Meniscus tear: pathology, incidence, and management. Cureus. 2022;14(5): e25121. 10.7759/cureus.25121 PMC9205760 35733484

[26] Mordecai SC Al-Hadithy N Ware HE Gupte CM. Treatment of meniscal tears: an evidence based approach. World J Orthop. 2014;5(3):233-241. 25035825 10.5312/wjo.v5.i3.233 PMC4095015

[27] Ogura T Suzuki M Sakuma Y , et al. Differences in levels of inflammatory mediators in meniscal and synovial tissue of patients with meniscal lesions. J Exp Orthop. 2016;3(1):7. 26915007 10.1186/s40634-016-0041-9 PMC4740471

[28] Previtali D Merli G Di Laura Frattura G Candrian C Zaffagnini S Filardo G. The long-lasting effects of “placebo injections” in knee osteoarthritis: a meta-analysis. Cartilage. 2021;13(1)(suppl):185S-196S. 10.1177/1947603520906597 PMC8808779 32186401

[29] Razi M Mortazavi SMJ . Save the meniscus, a good strategy to preserve the knee. Arch Bone Jt Surg. 2020;8(1):1-4. 32090138 10.22038/abjs.2019.45438.2242 PMC7007719

[30] Scanzello CR McKeon B Swaim BH , et al. Synovial inflammation in patients undergoing arthroscopic meniscectomy: molecular characterization and relationship to symptoms. Arthritis Rheum. 2011;63(2):391-400. 21279996 10.1002/art.30137 PMC3260472

[31] Seil R Becker R. Time for a paradigm change in meniscal repair: save the meniscus! Knee Surg Sports Traumatol Arthrosc. 2016;24(5):1421-1423. 27107860 10.1007/s00167-016-4127-9

[32] Waddell DD Bert JM. The use of hyaluronan after arthroscopic surgery of the knee. Arthroscopy. 2010;26(1):105-111. 20117634 10.1016/j.arthro.2009.05.009

