# Supplementary material for: Platelet-Rich Plasma Injections Do Not Improve the Recovery After Arthroscopic Partial Meniscectomy: A Double-Blind Randomized Controlled Trial
Source: Am J Sports Med. 2024 Oct 18;52(13):3198–205. doi: 10.1177/03635465241283052 (PMC11542319; doi:10.1177/03635465241283052)
Supplement: sj-pdf-1-ajs-10.1177_03635465241283052 – Supplemental material for Platelet-Rich Plasma Injections Do Not Improve the Recovery After Arthroscopic Partial Meniscectomy: A Double-Blind Randomized Controlled Trial [file sj-pdf-1-ajs-10.1177_03635465241283052.pdf]

Platelet-rich plasma injections do not improve the recovery after arthroscopic partial meniscectomy: A double-blind randomized controlled trial

Appendix Table A1. Subjective clinical scores at baseline and follow-ups in both groups.

Outcome	Group	Baseline	30 days	60 days	180 days	Anova test
**IKDC Subjective**	Treatment	47.4 ± 14.0	59.9 ± 16.1	70.8 ± 17.0	79.0 ± 14.9	p < 0.0005
	Control	49.7 ± 17.9	60.0 ± 15.9	75.6 ± 16.8	77.8 ± 17.1	p < 0.0005
**KOOS Pain**	Treatment	62.5 ± 18.3	77.3 ± 15.6	86.3 ± 14.9	92.9 ± 10.7	p < 0.0005
	Control	65.5 ± 20.3	79.8 ± 15.8	89.8 ± 14.5	91.8 ± 12.4	p < 0.0005
**KOOS Symptoms**	Treatment	66.2 ± 17.1	76.4 ± 18.8	84.3 ± 15.8	88.1 ± 12.7	p < 0.0005
	Control	69.0 ± 20.2	79.2 ± 15.2	89.1 ± 13.3	90.4 ± 13.0	p < 0.0005
**KOOS ADL**	Treatment	75.7 ± 16.9	84.4 ± 14.3	92.8 ± 11.1	96.9 ± 8.2	p < 0.0005
	Control	75.2 ± 22.6	85.2 ± 16.8	94.5 ± 12.0	96.7 ± 7.8	p < 0.0005
**KOOS Sport/Rec**	Treatment	49.0 ± 23.0	59.4 ± 24.4	69.3 ± 22.2*	82.1 ± 19.4	p < 0.0005
	Control	53.7 ± 24.1	60.5 ± 22.1	79.3 ± 21.7*	78.5 ± 24.4	p < 0.0005
**KOOS QoL**	Treatment	39.7 ± 21.2	57.8 ± 21.4	64.7 ± 25.7*	79.3 ± 23.8	p < 0.0005
	Control	39.8 ± 20.1	57.7 ± 23.4	76.6 ± 22.1*	80.8 ± 21.6	p < 0.0005
**EQ-VAS Score**	Treatment	78.3 ± 11.9	82.3 ± 8.1	85.1 ± 7.2	88.6 ± 9.2	p < 0.0005
	Control	80.0 ± 12.7	82.8 ± 11.4	85.5 ± 12.0	88.0 ± 11.3	p = 0.009
**Tegner Score**	Treatment	2.7 ± 1.3	2.9 ± 1.0	3.6 ± 1.2	4.3 ± 1.6	p < 0.0005
	Control	2.9 ± 1.7	3.2 ± 1.0	3.9 ± 1.4	3.9 ± 1.5	p < 0.0005

Both groups showed an improvement over time (Anova test). The comparison of the two groups showed only two differences: * $p < 0.05$ in favor of the control group. Values are expressed as mean ± standard deviation. ADL, Activities of Daily Living; EQ-VAS, EuroQol–visual analog scale; IKDC, International Knee Documentation Committee; KOOS, Knee injury and Osteoarthritis Outcome Score; QoL, quality of life, Sport/Rec, sport/recreation.
